# Its Utility in the Feeding of Infants

**Published:** 1920-09-18

**Authors:** 


### CONDENSED MILK.

### Its Utility in the Feeding of Infants.

A most interesting little treatise* on this subject, by Dr. P. Lassablière, has come to our notice, and in that it sets forth a number of plain facts which are of vital importance at a time when the condensed-milk problem is under official consideration (see p. 615), it is perhaps worth while to make particular note of some of the author's conclusions. Dr. Lassablière's sketch was awarded the "Prix Jeunesse, 1919," by the Paris Faculty of Medicine, and this reward alone gives authority to the summaries which we quote.

### Artificial v. Natural Feeding.

But in the first place there must be no misunderstanding of the moral intended. For the new-born infant any method of upbringing, except the natural one, at the breast, is to be condemned utterly. The cleverest chemists, were they a thousand or a hundred thousand, will not succeed in inventing for a new-born infant a food *superior* to human milk. Deliberately to replace the breast by a feeding-bottle or a spoon is incontestibly a crime. Whenever breast-feeding is possible, it should be enjoined vigorously and exclusively. Physiology and medical practice, supported by innumerable statistics and fortified by common-sense, demonstrate that for little children anything that is not maternal milk is only a second-best food substance.

Nevertheless, there are cases, unfortunately only too numerous, in which breast-feeding is impossible. The host of possible causes need no explanation here, and the question inevitably arises, which of the substitutes shall we choose? The author of this particular treatise sets forth a strong case for the well-known condensed milk. We will ourselves at least assert that it is in point of fact an excellent milk, which has not been heated beyond eighty degrees, to which the only foreign substance added is cane sugar, a chemically pure food, easy of digestion. Also adulteration need not be feared and bacterial fermentations are impossible. Let us quote directly, too, from Professor Richel's preface. "Remember that in immense Nature there is only one single substance which has been *designed* as a food. Mutton and beef are designed to be mutton and beef; clover and wheat, to be clover and wheat. Do not suppose that Nature created wheat to be eaten by man or clover to be eaten by sheep; it is only milk which by its original design is intended to be eaten and digested. The function of milk is to be a food. That is why it is the best food."

### History.

No narrative could be richer in instruction for the child-welfare worker, for any in fact whose duties include the supervision of infant health, than the history of preserved milk and its development. No food product has excited deeper prejudice, or has had more determined detractors. The earliest record is probably the work of Newton, who added 1 to 2 per cent. of sugar to the milk and recommended slowly evaporating it on a water-bath in a current of hot air, "thus producing a thick cream or a soft paste." Thence we pass on through a host of necessary and unnecessary innovations up to the well-known product of to-day. For the details for the use of condensed milk under conditions such as the Siege of Paris, and many other exacting circumstances, we must refer the reader to Dr. Lassablière's monograph, but suffice it to say that the paramount value of condensed milk as an emergency food product has been many times exemplified.

### The Hygienic Point of View.

By its very composition, milk is one of the most easily altered foods, nor is there a food more subject to frauds and falsifications. Bad milk is in any case worse than bad meat, not only because it is more insidious in its attacks, but also because it strikes at the source of mankind in its ill effects on the infantile population. Nowadays, in spite of the "clean milk" campaign, there is still a certain amount of ignorance prevalent in milk collection, and it should be known by all that milk once even lightly infected with micro-organisms, be they harmless or pathogenic, becomes within a very short time a culture simply swarming with bacteria of all kinds which have found themselves all in a medium highly favourable to their development. If we can guarantee a universal supply of clean milk the following suggestions would be unnecessary, but as things are we may well remember that "condensed milk escapes many criticisms." Aseptically collected and prepared, it commends itself to us by a perfect preservation and a constant composition. Enclosed in soldered tins it is exempt from every kind of fraud, and this offers absolute security to the consumer. It has stood its trial in hot countries and colonial expeditions. It satisfies all the requirements of infant feeding; and there is no more sensitive reagent than a new-born infant by which to test the value of a milk. Finally, even as compared with sterilised milk, it possesses the advantages of a reduced volume.

### The Physiological Standpoint.

To those who wish to pursue the physiological side of the question, it is explained that experimental addition of rennet to sweetened condensed milk such as is given to children, a liquid is obtained in which no separation occurs, even after two days. The casein appears to be in a form bordering on the colloidal, which is interesting evidence in view of the repeated discussions concerning the heaviness of the curd produced from the usual cow's milk. Two other points are made by Dr. Lassablière—namely, (*a*) that in spite of the large amount of saccharose

* Published under the auspices of the monthly review, *La Médecine*, 21 Boulevard St. Germain, Paris.

**Condensed Milk**—(*continued*).

taken with condensed milk, the children show no signs associated with excessive ingestion of sugar and no sugar is found in the urine, and that (*b*) the stools of children fed on condensed milk bear much more resemblance to those of children fed at the breast than to those of children fed on fresh cow's milk. Bacteriologically, too, the excess of sugar is not found to influence adversely the intestinal flora or their relative normal proportions. Add to this the fact that all the investigator's test cases *do* thrive on the condensed-milk feeding, and we see there is ample evidence for considering that condensed milk is really an invaluable asset in dealing with our infant-welfare problems.

The Clinical Aspect.

Clinically, however, the great difficulty has been the very different quality of the milks used and the different methods of using them, *i.e.*, the selected dilutions and size of feeds. "In this respect the indications furnished by the labels on the tins have been the cause of regrettable confusion and of unavoidable errors. These indications, in fact, vary greatly for the different brands, and are, for the most part, completely erroneous. But the danger which the commercial indications present may be avoided, provided the doctor (whose *rôle* it is) himself prescribes the exact quantity and the precise method of employment for each child." And in these words we have summed up the chief lesson which Dr. Lassablière's work provides. We feel sure that in this country, apart from meeting actual opposition, the principle of condensed-milk feeding will not gain a strong foothold, because in almost any circumstances other, at present more favoured, artificial products are so frequently upheld. But occasions have arisen and will arise where the successful use of condensed milk may solve many an urgent infantile problem, and to the doctor or nurse who would have the knowledge how correctly to use this food, we strongly recommend an understanding inspection of this short treatise from Paris. It is in many ways instructive.

